# Copy Number Variants and Genetic Polymorphisms in *TBX21*, *GATA3*, *Rorc*, *Foxp3* and Susceptibility to Behcet's Disease and Vogt-Koyanagi-Harada Syndrome

**DOI:** 10.1038/srep09511

**Published:** 2015-04-15

**Authors:** Dan Liao, Shengping Hou, Jun Zhang, Jing Fang, Yunjia Liu, Lin Bai, Qingfeng Cao, Aize Kijlstra, Peizeng Yang

**Affiliations:** 1 The First Affiliated Hospital of Chongqing Medical University, Chongqing Key Laboratory of Ophthalmology and Chongqing Eye Institute, Chongqing, China; 2 University Eye Clinic Maastricht, Maastricht, The Netherlands

## Abstract

This study aimed to investigate the role of genetic variants including single nucleotide polymorphisms (SNPs) and copy number variants (CNVs) of *TBX21*, *GATA3*, *Rorc* and *Foxp3* genes in Behcet's disease (BD) and Vogt-Koyanagi-Harada (VKH) syndrome in a Chinese Han population. Genotyping of 25 SNPs was performed by iPLEX system (Sequenom) or polymerase chain reaction-restriction fragment length polymorphism (PCR-RFLP). TaqMan real time PCR was used to assess CNVs. The expression of *Rorc* and *Foxp3* were examined by real-time PCR and cytokine production was measured by ELISA. High *Rorc* CNV was associated with the susceptibility to BD (P = 8.99 × 10^−8^, OR = 3.0), and low *Foxp3* CNV predisposed to BD in female patients (P = 1.92 × 10^−5^, OR = 3.1). CNVs for the investigated genes were not altered in VKH syndrome. Further functional studies demonstrated that the relative mRNA expression levels of *Rorc* were increased in individuals with high *Rorc* copy number, but not for *Foxp3*. Increased production of IL-1β and IL-6 was found in individuals carrying a high CNV of Rorc. Our study showed that high CNVs of *Rorc* and low CNVs of *Foxp3* confer risk for BD but not for VKH syndrome. The tested 25 SNPs in *TBX21*, *GATA3*, *Rorc* and *Foxp3* did not associate with BD and VKH syndrome.

Inflammation of the uvea, a highly vascularized and pigmented area, is called uveitis. It is a relatively common eye disease^1^ as well as one of the main causes of blindness in the world^2^. Behcet's disease (BD) and Vogt-Koyanagi-Harada (VKH) syndrome are two uveitis entities commonly seen in Asia^3^,^4^. However, these diseases display marked differences in their pathogenesis and clinical features. BD appears as a chronic systemic inflammatory disorder with a diverse spectrum of clinical manifestations including recurrent uveitis, oral aphthae, genital ulcers and skin lesions^5^. VKH syndrome on the other hand is a multi-systemic autoimmune disease featuring bilateral granulomatous panuveitis associated with poliosis, vitiligo, alopecia, auditory and central nervous system signs^6^. Although the etiology of uveitis remains unclear, genetic variants of immune related genes are considered to be important contributors to this disease as evidenced by a strong association with immune genes including genes encoding for the HLA system and various non-HLA genes such as *IL23R* and *IL10*^7^,^8^.

The immune system is poised in a state of equilibrium. Immune or inflammatory disorders are characterized by a critical imbalance in T lymphocyte function and CD4^+^ T cells are central for the homeostasis^9^,^10^. CD4^+^ T cells are generally considered as having a pre-defined role as helper T cells within the immune system. Accumulative data showed that pathogenic CD4^+^ T cells are recognized as critical contributors to the development of Behcet's disease and VKH syndrome as evidenced by the aberrant production of Th1, Th2, Th17 and Treg cytokines and the aberrant frequency of these four T cell subsets^11^,^12^,^13^,^14^,^15^,^16^,^17^,^18^,^19^. *TBX21*, *GATA3*, *Rorc* and *Foxp3* are key transcription factors for CD4^+^ T cell subsets including Th1, Th2, Th17 and Treg cells, respectively. Previous studies found the dysregulations of *TBX21/GATA3* and *Rorc/Foxp3* ratios in uveitis patients with neuro-BD^20^, suggesting that a dysregulated function of these T cell subsets may lead to immune imbalance, proliferation and activation of pathogenic CD4^+^ T cells subsequently leading to uveitis.

*Foxp3* polymorphisms were found to be associated with multiple autoimmune disorders such as type 1 diabetes, Graves' disease and psoriasis^21^,^22^,^23^,^24^. Additionally, certain SNPs in *Foxp3* were found to significantly enhance transcription activity and lead to higher mRNA levels of *Foxp3*, suggesting that the SNP in the *Foxp3* gene may have functional effects on immune tolerance mediated by Treg cells^25^. Previous studies also showed the association of genetic polymorphisms in *TBX21* with systemic lupus erythematosus, type 1 diabetes, systemic sclerosis and Graves' disease^26^,^27^,^28^,^29^.

However, the association of these four transcriptional factors with BD and VKH syndrome has not yet been addressed and was therefore the aim of our study whereby we explored the association of two genetic variants including CNVs as well as SNPs of *TBX21*, *GATA3*, *Rorc* and *Foxp3* in the pathogenesis of uveitis via a two-stage case control study. The results showed that a high CNV of *Rorc* and a low CNV for *Foxp3* were associated with the susceptibility with BD but not with VKH syndrome in a Chinese Han population. No association was found for the tested 25 SNPs of the four genes with BD and VKH syndrome.

## Results

### Clinical Features of BD and VKH Syndrome Patients

The clinical findings of the BD and VKH patients enrolled in our study are shown in Supplementary Table 1–2.

### Allele and Genotype Frequencies of Selected SNPs in Patients and Normal Controls

We applied the iPLEX system (Sequenom) and PCR-RFLP measurements to successfully evaluate 25 SNPs at *TBX21*, *GATA3*, *Rorc* and *Foxp3* genes in an independent cohort containing 406 BD patients, 401 VKH syndrome patients and 613 healthy controls. The frequencies of genotype and allele of 22 SNPs at *TBX21*, *GATA3*, *Rorc* genes didn't show a significant difference among BD patients, VKH syndrome patients and healthy controls (Supplementary Table 3). Considering that *Foxp3* is X-linked, we split each study colony into two groups by gender. After stratification by gender, no association was found for three SNPs in the *Foxp3* gene with BD or VKH syndrome (Supplementary Tables 4 and 5).

### First Stage Study for Copy Number Variations of *TBX21*, *GATA3, Rorc* and *Foxp3* in Behcet's Disease and VKH Syndrome

A total of 406 BD patients, 401 VKH syndrome patients and 613 healthy controls were enrolled in the first stage CNV study. An increased frequency of high *Rorc* copy number was observed in patients with BD, but not in VKH patients (P = 0.001, OR = 2.7, 95% CI 1.5–5.1) (Table 1). A reduced *Foxp3* CNV was found in female BD patients (P = 9.85 × 10^−4^, OR = 4.1, 95% CI 1.7–10.1) (Table 2). The result showed that no association of *Foxp3* CNVs with VKH syndrome. Additionally, no significant difference was found for the CNVs of *TBX21* and *GATA3*.

### The Replication and Combining Studies for Copy Number Variants of *Rorc* and *Foxp3* in Behcet's Patients and Controls

To further verify the association of *Rorc* and *Foxp3* with BD, an additional set of 642 BD patients and 1623 healthy controls were evaluated for *Rorc* and *Foxp3* CNVs (second stage or replication study). The replication results showed that a high *Rorc* CNV was consistently linked to the susceptibility to BD (P = 1.44 × 10^−4^, OR = 2.9, 95% CI 1.6–5.1) (Table 1). Reduced *Foxp3* CNV again predisposed to BD in females (P = 2.36 × 10^−3^, OR = 2.7, 95% CI 1.4–5.4) (Table 2). Combining the two stage data showed that a high *Rorc* CNV (>2) was associated with the susceptibility to BD (P = 8.99 × 10^−8^, OR = 3.0, 95% CI 2.0–4.6) (Table 1). A low *Foxp3* CNV (<2) predisposed to BD in females (p = 1.92 × 10^−5^, OR = 3.1, 95% CI 1.8–5.2) (Table 2) and a high *Foxp3* (>2) CNV was a protective factor for BD in males (P = 5.84 × 10^−4^, OR = 0.5, 95% CI 0.3–0.7) (Table 3).

### Association between *Rorc/Foxp3* Copy Number Variants and mRNA Levels

**Figure 1:**
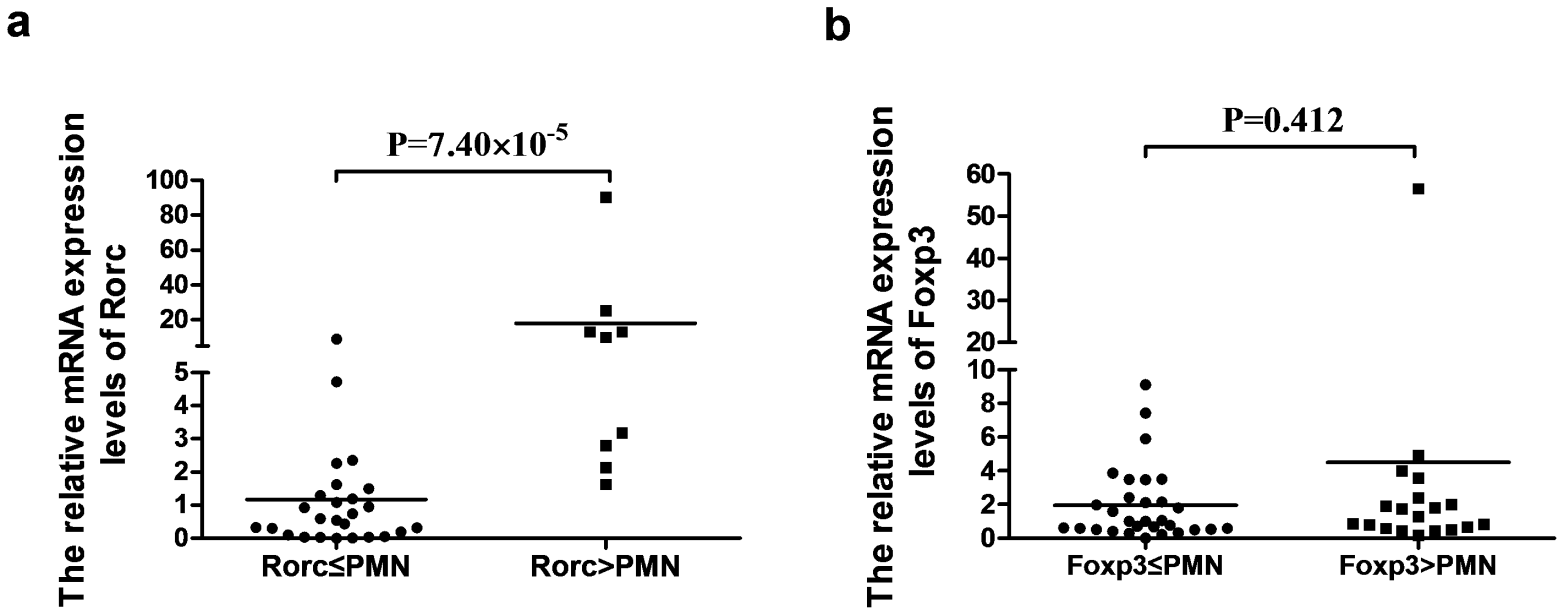
The Influence of CNVs in Rorc/Foxp3 on the mRNA Levels. (a) The mRNA expression of Rorc in PBMCs from controls carrying different copies of gene (Rorc ≤ PMN: n = 26, Rorc > PMN: n = 9). (b) The mRNA expression of Foxp3 in PBMCs from controls carrying different copies of gene (Foxp3 ≤ PMN: n = 30, Foxp3 > PMN: n = 19). Significance was examined using SPSS's two independent samples Nonparametric test. PMN represents the population specific median copy number.

Because a gene dosage effect may be one of the mechanisms of action for CNVs, we investigated the association between *Rorc/Foxp3* copy number variations and mRNA expression levels in healthy individuals. The expression studies of *Rorc* and *Foxp3* were performed using samples from healthy controls (cDNA bank), since the patients enrolled in this study represented a heterogeneous group of individuals with marked differences in the degree of immunosuppressive therapy received and inflammation response. The relative mRNA expression levels of *Rorc* were increased in individuals with high Rorc copy number (Fig. 1a). For *Rorc*, the population specific median copy number (PMN) is two. The PMN of *Foxp3* in female is two while the PMN of *Foxp3* in male is one. A significantly up-regulated expression of *Rorc* was found in the high copy group (*Rorc* > PMN) when compared to the low copy number group (*Rorc* ≤ PMN) (P = 7.40 × 10^−5^). To further determine the relationship between Rorc mRNA expression and its CNV, correlation analysis was performed. The results showed a positive association between Rorc mRNA expression with its CNV (P = 0.04, r^2^ = 0.349) (Supplementary Fig. 1). However, no significant difference was found between *Foxp3* copy number variations and mRNA levels (Fig. 1b).

### The Influence of *Rorc* Copy Number Variants on Cytokine Production

**Figure 2:**
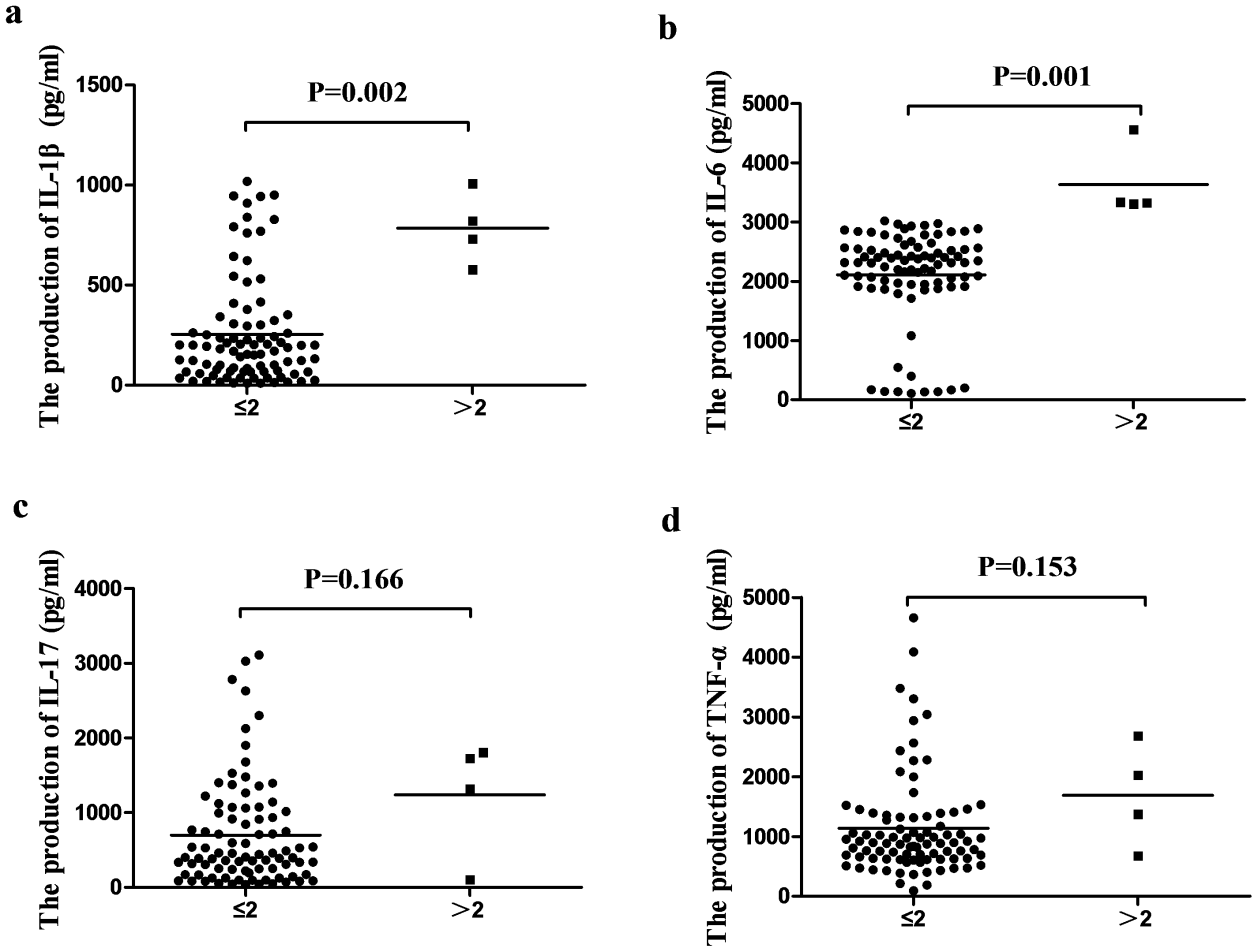
The Influence of CNVs in Rorc on Cytokine Production. The production of IL-1β(a), IL-6 (b), IL-17(c) and TNF-α(d) by stimulated PBMCs from healthy controls carrying different copy number of Rorc (Rorc ≤ 2: n = 88, Rorc > 2: n = 4). Significance was examined using SPSS's two independent samples Nonparametric test.

The results of RT-PCR showed that CNVs of *Rorc* had an effect on its transcriptional regulation. IL-1β, IL-6, IL-17 and TNF-α-were shown to be involved in the development of Behcet's disease^30^,^31^,^32^,^33^,^34^. Further experiments were therefore performed to investigate whether *Rorc* CNVs affected the production of these four cytokines. As IL-1β is produced by activated macrophages, rather than specific subsets of T cells^35^,^36^, we tested the production of these cytokines using PBMCs that generally include a certain number of macrophages. The results showed that an increased production of IL-1β (P = 0.002) and IL-6 (P = 0.001) by stimulated PBMCs was discovered in individuals carrying a high CNV of *Rorc* (Fig. 2a–b). No significant association was found between IL-17 and TNF-α by stimulated PBMCs and CNV of *Rorc* (Fig. 2c–d).

## Discussion

In this study we show that the frequency of high *Rorc* CNV was significantly increased in BD, while an increased frequency of low *Foxp3* CNV was found in female BD patients. Further functional studies demonstrated that the relative mRNA expression levels of *Rorc* were increased in individuals with high *Rorc* copy number.

Our data suggest a ratio dysregulation of *Rorc/Foxp3* in BD patients which is in accordance with a previous report in neuro-BD patients^20^. Previous studies have investigated the association of CNVs at C4, IL17F, IL23A and DEFA1 with BD^37^,^38^,^39^. The present study identified two additional risk CNVs with BD including *Rorc* and *Foxp3*. The present study also examined the association of CNVs of *Rorc* and *Foxp3* with another uveitis entity, VKH syndrome. However, no association was found for this syndrome. The discrepancy between *Rorc* and *Foxp3* CNVs and susceptibility to these two uveitis entities may be due to the different features of these two diseases. Behcet's disease is considered as a non-granulomatous inflammation^40^, whereas VKH syndrome is in fact a granulomatous inflammation^41^,^42^, suggesting that *Rorc* and *Foxp3* may be involved in these two diseases via different mechanisms.

TBX21, GATA3, Rorc and Fxop3 are classical master regulators of CD4^+^ helper T cell polarization and function. Our results showed that the genetic variants at *Rorc* and *Foxp3,* rather than *TBX21*, *GATA3*, are involved in the development of BD, suggesting CD4^+^ helper T cell subgroups contribute to this disease via a different pathway. *Rorc* and *Foxp3* are key transcription factors for Th17 and Treg cells which have inverse impacts on autoimmunity. Th17 cells mainly regulated by *Rorc* produce IL-17, TNF-α and IL-6 to promote the development of autoimmunity and allergic reactions^43^. However, Treg cells mainly regulated by *Foxp3* express anti-inflammatory cytokines such as IL-10 and TGF-β1, suggesting that they play an anti-inflammatory role and maintain immunologic tolerance to self-antigens^44^. The absence of Treg cells in *Foxp3* mutant individuals could result in a breakdown of immunologic tolerance and uncontrolled inflammatory responses^45^. The Th17/Treg balance thus contributes to maintain a proper immune homeostasis. Our results also showed an increased production of IL-1β and IL-6 by stimulated PBMCs in individuals carrying a high CNV of *Rorc*. IL-6 and IL-1β are critical regulators of Th17 differentiation and are seen as inflammatory markers. Th17 cells are characterized by the secretion of IL-17 in addition to other pro-inflammatory cytokines such as TNF-α^46^. Increased expression of Rorc, IL-6 and IL-1β in psoriatic skin has been reported indicating their participation in the pathogenesis of immune disease^47^. Moreover, a higher increase of IL-6 has been previously reported in active BD patients^48^.

As one type of the most common genetic variations in the human genome, SNPs are concerned with human phenotypic differences and the susceptibility to disease^49^. In the present study, we selected 25 tagSNPs of *TBX21*, *GATA3*, *Rorc* and *Foxp3*. Contrary to previous studies that showed evidence of disease association in Caucasian populations^21^,^22^,^23^,^24^, no association was found between these 25 SNPs and the susceptibility to BD or VKH syndrome in Han Chinese. Composite analysis of our genotype data and HapMap data for Caucasian population showed that 18 out of 25 tested SNPs in this present study were significantly different concerning their allele frequency when comparing Han Chinese with Caucasians (Supplementary Table 6). Moreover, our recent Genome-wide association study didn't show an association between these 25 SNPs and risk of Behcet's disease and VKH syndrome^50^,^51^, suggesting that the SNPs at these four genes may be not involved in these two uveitis entities in Chinese Han. The inconsistent results between Caucasian and Han Chinese populations indicate a genetic heterogeneity towards disease susceptibility.

Our data suggest that a high gene copy number of *Rorc* may play a role in BD pathogenesis via the transcriptional regulation as well as a change in protein activity/expression. Further analysis of the biological function in individuals carrying a high CNV of *Rorc* is hampered by the fact that the frequency is as low as 1.70% and therefore only 4 samples with *Rorc* > 2 were enrolled in this study. We furthermore failed to find how the CNVs of *Foxp3* exactly affect the development of BD. Further functional studies are therefore needed to elucidate the exact role of *Rorc* and *Foxp3* genes in the development of BD. The role of gender in the predisposition to BD and the fact that a low Foxp3 CNV predisposed to BD in females requires further analysis. Other limitations of our study include the fact that patients were recruited from ophthalmic centers and may thus represent a selected population of patients. Further confirmation is needed by investigating BD patients from other medical specialties such as rheumatology or dermatology departments.

In conclusion, high *Rorc* CNV and low *Foxp3* CNV were associated with the susceptibility to BD in a Chinese Han population. No association was found for the 25 SNPs at *TBX21*, *GATA3*, *Rorc* and *Foxp3* genes with BD and VKH syndrome.

## Methods

### Study Population

The study group comprised 1048 BD patients and 401VKH syndrome patients who all belong to Chinese Han descent and were recruited from the First Affiliated Hospital of Chongqing Medical University (Chongqing, China) or the Zhongshan Ophthalmic Center of Sun Yat-sen University (Guangzhou, China) between October 2005 and January 2014. A total of 2236 unrelated, unselected normal controls were age-, ethnicity- and geography-matched with the patients. The criteria of the International Study Group for BD and First International Workshop for VKH were set as the diagnostic criteria for the diagnosis of BD and VKH syndrome respectively^52^,^53^. If there was any doubt in the diagnosis, the patients were excluded from the study.

### Ethics Statement

All venous blood samples were collected after approval from the relevant research ethics committees and written informed consent was obtained from all the participants. The tenets of the Declaration of Helsinki were upheld during all procedures of this study. The present study was approved by the Clinical Research Ethics Committee of the First Affiliated Hospital of Chongqing Medical University (Permit Number: 2009-201008). All methods were performed in accordance with the approved guidelines.

### SNPs Selection

The SNPs examined in this study were tagSNPs chosen from HapMap data using Chinese Han Beijing data (MAF ≥ 0.05). As a result, a total of 25 SNPs were genotyped and analyzed in this study (Supplementary Table 3–5).

### SNP Genotyping

Genomic DNA was extracted from peripheral blood of patients and controls using the QIAamp DNA Blood Mini Kit (QIAGEN Inc., Hilden, Germany) strictly following the manufacturer's instructions. Genotyping of 19 SNPs was determined by using the iPLEX system (Sequenom) in MassARRAY® platform according to the manufacturer's instructions. The primers to genotype these 19 SNPs were designed by MassARRAY Assay design software (Sequenom) (Supplementary Table 7). Genotyping of another 6 SNPs was performed by polymerase chain reaction-restriction fragment length polymorphism (PCR-RFLP). The primers and restriction enzymes for PCR-RFLP were seen on Supplementary Table 8. Direct sequencing was performed in 5% of the total samples randomly chosen in order to validate the method employed in this study. All SNPs tested in this study had a genotyping success rate >95% and accuracy >99% in all subjects.

### Copy Number Genotyping

For accurate quantification of each CNV, we have developed a corresponding TaqMan real time PCR assay performed in 96-well optical plates on a 7500 real-time PCR system following the manufacturer's protocols (Applied Biosystems, Foster City, CA). TaqMan assays labeled with FAM were used to detect *TBX21* (Hs06419992-cn), *GATA3* (Hs02236731-cn), *RORC* (Hs00427550-cn) and *Foxp3* (Hs02146785-cn) respectively (Applied Biosystems, Foster City, CA). TaqMan® RNaseP assay labeled with VIC was used as an internal copy number reference (Applied Biosystems, Foster City, CA). The amplification conditions were: 10 min at 95°C and 40 cycles of 95°C for 15 sec and 60°C for 1 min. In a model experiment, the method was shown to accurately quantify targets that vary in the range from 0 to 5 copies per genome. All samples were analyzed in triplicate.

### Cell Culture and Cytokine Measurements

Peripheral blood mononuclear cells (PBMCs) were isolated from venous blood using Ficoll-Hypaque density-gradient centrifugation. Cell culture medium consisted of RPMI 1640 complete medium, 10% fetal calf serum, 2 mML-glutamine and 100 U/ml penicillin/streptomycin(Invitrogen, Carlsbad, CA). PBMCs were stimulated with LPS (100 ng/ml, Sigma, Missouri, USA) for 24 h to detect TNF-α, IL-1β, IL-6 at a density of 1 × 10^6^ cells/mL. For IL-17 detection, PBMCs were stimulated with anti-CD3 (OKT3, 0.5 μg/ml) plus anti-CD28 antibodies (15E8, 0.1 μg/ml) (Miltenyi Biotec, Palo Alto, CA) for 72 h. The concentration of tumor necrosis factor α (TNF-α), IL-1β, IL-6 and IL-17 in cell culture supernatants was examined with DuosetELISA development kits (R&D Systems, Minneapolis, MN).

### Real-time Quantitative PCR Analysis

Total RNA was isolated with Trizol reagent (Invitrogen, Carlsbad, CA, USA) and reverse transcribed with the Prime Script RT reagent Kit (TaKaRa, Dalian, China) strictly following the manufacturer's instructions. The real-time quantitative PCR (RT-PCR) was performed using the ABI7500 Fast System(Applied Biosystems, Foster City, CA).The primers used for *β-actin*, *Rorc* and *Foxp3* mRNA detection were as follows: *β-actin*F: 5′GGATGCAGAAGGAGATCACTG3′ and *β-actin*R: 5′CGATCCACACGGAGTACTT3′; *Rorc*F: 5′AGAAGACCCACACCTCACAA3′ and *Rorc*R: 5′CCTCACAGGTGATAACCCCG3′; *Foxp3*F: 5′ATTCCCTGAGCCCTGATCCA3′ and *Foxp3*R: 5′TGGGGAGCTCGGCTG3′. The relative expression levels of target genes were normalized to the expression of internal reference gene *β-actin* using the 2^−CT^ method.

### Statistical Analysis

For SNP analysis, Hardy–Weinberg equilibrium (HWE) was tested using the Chi-square test. Genotype frequencies were estimated by direct counting. Allele and genotype frequencies were compared between patients and controls by the chi-square test using SPSS version 17.0. As to the CNV analysis, relative quantitation analysis was performed using CopyCaller, v2.0 (Applied Biosystems, Foster City, CA).Statistical comparison between the patients and controls was performed by Chi-square test using SPSS. The P values were corrected (Pc) for multiple comparisons with the Bonferroni correction. Cytokine expression and RT-PCR data were analyzed by two independent samples nonparametric test. P values less than 0.05 were considered statistically significant.

## Author Contributions

D.L., S.H. and P.Y. conceived the idea. D.L. and S.H. designed the experiments, J.Z., Y.L. and L.B. performed the experiments, J.F. and Q.C. analyzed the data. D.L., A.K. and P.Y. wrote the manuscript. S.H. prepared figures. All authors reviewed the manuscript.

## Supplementary Material

Supplementary Information Supplementary Information

## Figures and Tables

**Table 1:** The association of Copy number variant of TBX21, GATA-3, Rorc and Foxp3 with Behcet's disease and VKH syndrome

Genes	Stage	CNVs	BD (frequency)	VKH (frequency)	Control (frequency)	BD P	OR (95% CI)	VKH P	OR (95% CI)
TBX21	Stage 1	<2	44 (11.1)	44 (11.0)	51 (8.6)	0.181	1.3 (0.9–2.0)	0.194	1.3 (0.9–2.0)
		= 2	231 (58.3)	249 (62.4)	364 (61.1)	0.388	0.9 (0.7–1.2)	0.672	1.1 (0.8–1.4)
		>2	121 (30.6)	106 (26.6)	181 (30.4)	0.950	1.0 (0.8–1.3)	0.194	0.8 (0.6–1.1)
GATA3	Stage 1	<2	12 (3.0)	23 (5.8)	17 (2.8)	0.801	1.1 (0.5–2.3)	0.533	1.2 (0.7–2.3)
		= 2	354 (89.8)	346 (87.4)	571 (93.1)	0.062	0.7 (0.4–1.0)	0.007	0.6 (0.4–0.9)
		>2	28 (7.1)	27 (6.8)	25 (4.1)	0.036	1.8 (1.0–3.1)	0.096	1.6 (1.1–2.8)
Rorc	Stage 1	<2	8 (2.0)	12 (3.1)	22 (3.6)	0.152	0.6 (0.2–1.3)	0.711	0.9 (0.4–1.8)
		= 2	361 (91.2)	347 (91.1)	575 (93.8)	0.114	0.7 (0.4–1.1)	0.107	0.7 (0.4–1.1)
		>2	27 (6.8)	22 (5.8)	16 (2.6)	**0.001**	**2.7 (1.5–5.1)**	0.011	2.3 (1.2**-**4.4)
	Stage 2	<2	13 (2.0)	-	36 (2.2)	0.729	0.9 (0.5**-**1.7)	-	-
		= 2	613 (94.0)	-	1557 (96.3)	0.013	0.6 (0.4**-**0.9)	-	-
		>2	26 (4.0)	-	23 (1.4)	**1.44 × 10^−4^**	**2.9 (1.6–5.1)**	-	-
	Combined	<2	21 (2.0)	-	58 (2.6)	0.298	0.8 (0.5–1.3)	-	-
		= 2	974 (92.9)	-	2132 (95.6)	0.001	0.6 (0.4–0.8)	-	-
		>2	53 (5.1)	-	39 (1.7)	**8.99 × 10^−8^**	**3.0 (2.0–4.6)**	-	-

Bonferroni correction for the number of CNVs tested by the conditional analysis, P value less than 0.05/32 = 0.0016 was supposed significant.

**Table 2:** The association of Copy number variant of Foxp3 with Behcet's disease and VKH syndrome in female

Genes	Stage	CNVs	BD (frequency)	VKH (frequency)	Control (frequency)	BD P	OR (95% CI)	VKH P	OR (95% CI)
Foxp3	Stage 1	<2	9 (17.3)	7 (4.0)	14 (5.1)	**9.85 × 10^−4^**	**4.1 (1.7–10.1)**	0.613	0.8 (0.3–2.0)
		= 2	41 (78.8)	155 (89.1)	257 (92.8)	1.60 × 10^−3^	0.3 (0.1–0.7)	0.174	0.6 (0.3–1.2)
		>2	2 (3.8)	12 (6.9)	6 (2.2)	0.47	1.8 (0.4–9.2)	0.012	3.3 (1.2–9.1)
	Stage 2	<2	13 (12.5)		34 (4.9)	**2.36 × 10^−3^**	**2.7 (1.4–5.4)**	-	-
		= 2	87 (83.7)		621 (90.3)	0.041	0.6 (0.3–1.0)	-	-
		>2	4 (3.8)		33 (4.8)	0.669	0.8 (0.3–2.3)	-	-
	Combined	<2	22 (14.0)		49 (5.1)	**1.92 × 10^−5^**	**3.1 (1.8–5.2)**	-	-
		= 2	129 (82.2)		879 (90.9)	8.48 × 10^−4^	0.5 (0.3–0.7)	-	-
		>2	6 (3.8)		39 (4.0)	0.900	0.9 (0.4–2.3)	-	-

Bonferroni correction for the number of CNVs tested by the conditional analysis, P value less than 0.05/32 = 0.0016 was supposed significant.

**Table 3:** The association of Copy number variant of Foxp3 with Behcet's disease and VKH syndrome in male

Genes	Stage	CNVs	BD (frequency)	VKH (frequency)	Control (frequency)	BD P	OR (95% CI)	VKHP	OR (95% CI)
Foxp3	Stage 1	0	1 (0.3)	0	0	-	-	-	-
		1	333 (97.1)	195 (93.8)	307 (93.0)	0.015	2.5 (1.2–5.3)	0.745	1.1 (0.6–2.3)
		>1	9 (2.6)	13 (6.3)	23 (7.0)	0.008	0.4 (0.2–0.8)	0.745	0.9 (0.4–1.8)
	Stage 2	0	2 (0.4)		2 (0.2)	0.569	1.8 (0.2–12.5)		
		1	528 (96.7)		902 (94.3)	0.034	1.8 (1.0–3.1)		
		>1	16 (2.9)		53 (5.5)	0.020	0.5 (0.3–0.9)		
	Combined	0	3 (0.3)		2 (0.2)	0.390	2.2 (0.4–12.9)		
		1	861 (96.9)		1191 (93.9)	0.002	2.0 (1.3–3.1)		
		>1	25 (2.8)		76 (6.0)	**5.84 × 10^−4^**	**0.5 (0.3–0.7)**		

Bonferroni correction for the number of CNVs tested by the conditional analysis, P value less than 0.05/32 = 0.0016 was supposed significant.
