# Supplementary material for: Copy Number Variants and Genetic Polymorphisms in TBX21, GATA3, Rorc, Foxp3 and Susceptibility to Behcet's Disease and Vogt-Koyanagi-Harada Syndrome
Source: Sci Rep. 2015 Apr 15;5:9511. doi: 10.1038/srep09511 (PMC4397537; doi:10.1038/srep09511)
Supplement: Supplementary Information [file srep09511-s1.doc]

**Copy Number Variants and Genetic Polymorphisms in *TBX21*, *GATA3*, *Rorc*, *Foxp3* and Susceptibility to Behcet’s Disease and Vogt-Koyanagi-Harada Syndrome**

Dan Liao, Shengping Hou, Jun Zhang, Jing Fang, Yunjia Liu, Lin Bai, Qingfeng Cao, Aize Kijlstra and Peizeng Yang

**Supplementary Figure 1**

**
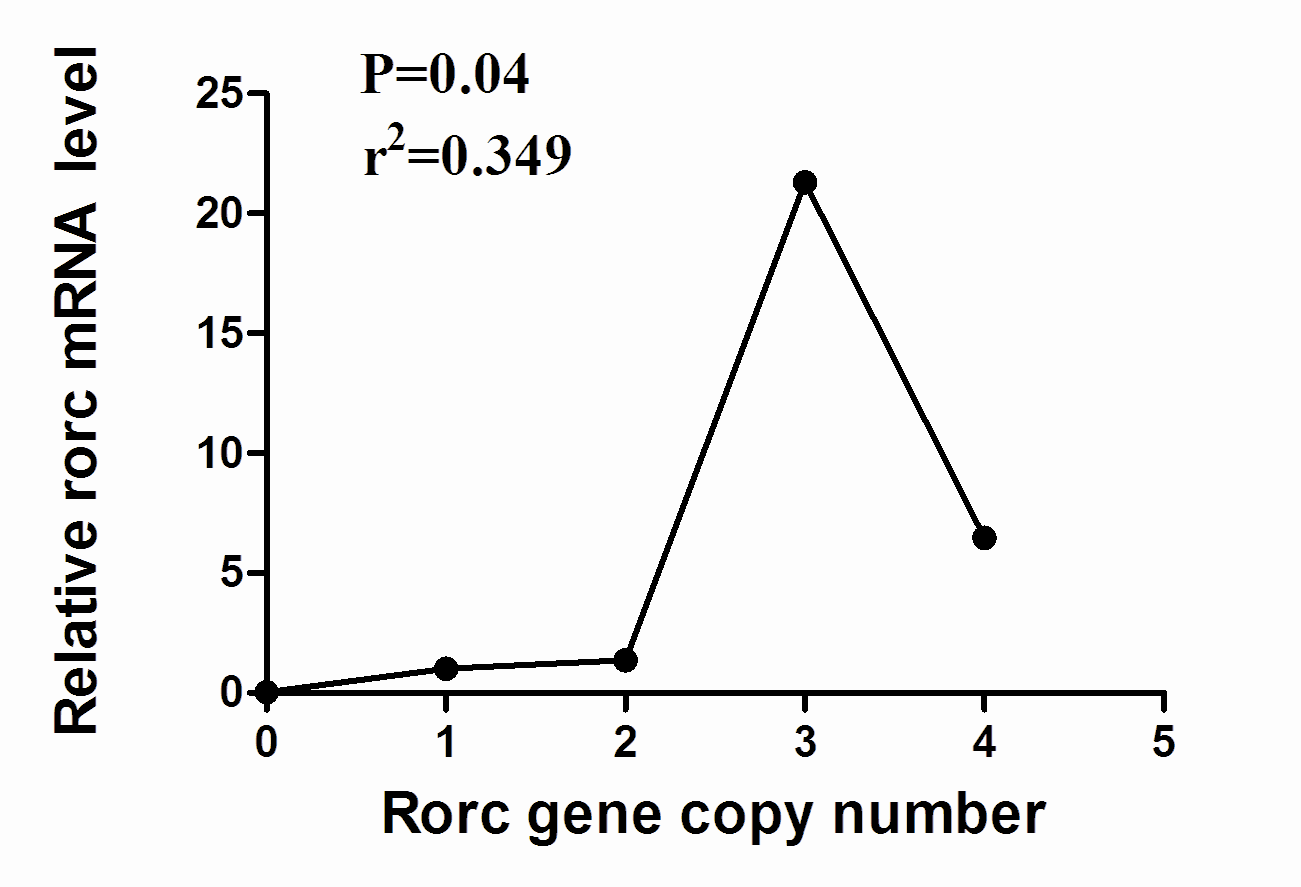
**

**Supplementary Figure Legends**

Correlation analysis demonstrates Rorc mRNA levels are increased in individuals with high Rorc copy number. Samples of 1 controls with Rorc gene deletion, 11 controls with one Rorc CN, 14 controls with two Rorc CN, 7 controls with three Rorc CN and 2 controls with four Rorc CN, were collected to measure serum levels of C4. Significance was examined using SPSS’s Bivariate Correlation.

**Supplementary Table 1 Clinical fingdings of Behcet’s patients enrolled in the present study**

| **Clinical features** | **Behcet’s Patients** | |
| --- | --- | --- |
|  | **N =1048** | **%** |
| Uveitis | 1048 | 100 |
| Oral ulcer | 1048 | 100 |
| Skin lesions | 733 | 69.9 |
| Genital ulcer | 586 | 55.9 |
| Arthritis | 157 | 15.0 |
| Positive pathergy test | 249 | 23.8 |
| Hypopyon | 304 | 29.0 |

**Supplementary Table 2** The clinical findings of VKH patients in this study

| **Clinical features** | **Number** | **Frequency** |
| --- | --- | --- |
|  | **N=401** | **%** |
| Uveitis | 401 | 100 |
| Nuchal rigidity | 73 | 18.2 |
| Headache | 132 | 32.9 |
| Scalp allergy | 64 | 16.0 |
| Tinnitus | 168 | 41.9 |
| Dysacusia | 99 | 24.7 |
| Alopecia | 136 | 33.9 |
| Poliosis | 137 | 34.2 |
| Vitiligo | 72 | 18.0 |

**Supplementary Table 3 Summary of the association of TBX21*,* GATA3andRorcSNPs with Behcet’s disease, VKH syndrome** in Chinese Han population

| **Genes** | **SNPs** | **Allele/Geno** | **BD**  **(frequency)** | **VKH (frequency)** | **Control**  **(frequency)** | **BD**  **P value** | **OR (95%CI)** | **VKH**  **P value** | **OR (95%CI)** |
| --- | --- | --- | --- | --- | --- | --- | --- | --- | --- |
| TBX21 | rs7502875 | C | 49 (6.1) | 42 (5.2) | 68 (5.7) | 0.719 | 1.1 (0.7-1.6) | 0.660 | 0.9 (0.6-1.4) |
|  |  | AA | 354 (87.8) | 360 (89.8) | 534 (89.4) | 0.430 | 0.9 (0.6-1.3) | 0.868 | 1.0 (0.7-1.6) |
|  |  | AC | 49 (12.2) | 40 (10.0) | 58 (9.7) | 0.220 | 1.3 (0.9-1.9) | 0.892 | 1.0 (0.7-1.6) |
|  |  | CC | 0 | 1 (0.2) | 5 (0.8) | ---- | --- | 0.411 | 0.3 (0.03-2.5) |
|  | rs10514934 | C | 96 (13.4) | 113 (14.1) | 150 (13.1) | 0.866 | 1.0 (0.8-1.3) | 0.545 | 1.1 (0.8-1.4) |
|  |  | CC | 7 (2.0) | 13 (3.1) | 8 (1.4) | 0.514 | 1.4 (0.5-3.9) | 0.052 | 2.4 (0.9-5.7) |
|  |  | CT | 82 (22.9) | 87 (21.7) | 134 (23.5) | 0.843 | 1.0 (0.7-1.3) | 0.516 | 0.9 (0.7-1.2) |
|  |  | TT | 269 (75.1) | 301 (75.2) | 429 (75.1) | 0.998 | 1.0 (0.7-1.4) | 0.974 | 1.0 (0.7-1.4) |
|  | rs16947058 | A | 144 (17.9) | 155 (19.5) | 230 (19.4) | 0.382 | 0.9 (0.7-1.1) | 0.980 | 1.0 (0.7-1.3) |
|  |  | AA | 17 (4.2) | 18 (4.5) | 20 (3.4) | 0.492 | 1.3 (0.7-2.4) | 0.358 | 1.4 (0.7-2.6) |
|  |  | AG | 110 (27.3) | 119 (29.9) | 190 (32.1) | 0.105 | 0.8 (0.6-1.1) | 0.069 | 1.3 (1.0-1.7) |
|  |  | GG | 276 (68.5) | 261 (65.6) | 382 (64.5) | 0.195 | 1.2 (0.9-1.6) | 0.734 | 1.0 (0.8-1.4) |
|  | rs2074190 | C | 91 (11.3) | 69 (8.6) | 117 (9.9) | 0.266 | 1.2 (0.9-1.7) | 0.360 | 0.9 (0.6-1.2) |
|  |  | TT | 319 (79.4) | 333 (83.5) | 487 (82.1) | 0.274 | 0.8 (0.6-1.2) | 0.586 | 1.1 (0.8-1.5) |
|  |  | CT | 75 (18.7) | 63 (15.8) | 95 (16.0) | 0.278 | 1.2 (0.9-1.7) | 0.922 | 1.0 (0.7-1.4) |
|  |  | CC | 8 (2.0) | 3 (0.8) | 11 (1.9) | 0.879 | 1.1 (0.4-2.7) | 0.178 | 0.4 (0.1-1.4) |

**Supplementary Table 3 (continued 1)**

| **Genes** | **SNPs** | **Allele/Geno** | **BD**  **(frequency)** | **VKH (frequency)** | **Control**  **(frequency)** | **BD**  **P value** | **OR (95%CI)** | **VKH**  **P value** | **OR (95%CI)** |
| --- | --- | --- | --- | --- | --- | --- | --- | --- | --- |
| TBX21 | rs4794067 | C | 108 (13.4) | 87 (10.9) | 84 (12.1) | 0.346 | 1.1 (0.9-1.5) | 0.452 | 0.9 (0.7-1.2) |
|  |  | TT | 303 (75.2) | 319 (79.7) | 464 (78.2) | 0.260 | 0.8 (0.6-1.1) | 0.569 | 1.1 (0.8-1.5) |
|  |  | CT | 92 (22.8) | 75 (18.8) | 116 (19.6) | 0.213 | 1.2 (0.9-1.7) | 0.750 | 0.9 (0.7-1.3) |
|  |  | CC | 8 (2.0) | 6 (1.5) | 13 (2.2) | 0.823 | 0.9 (0.4-2.2) | 0.435 | 0.7 (0.3-1.8) |
|  | rs17250932 | C | 65 (8.1) | 54 (6.8) | 90 (7.6) | 0.655 | 1.1 (0.8-1.5) | 0.438 | 0.9 (0.6-1.3) |
|  |  | TT | 338 (83.9) | 347 (87.0) | 508 (85.8) | 0.400 | 0.9 (0.6-1.2) | 0.604 | 1.1 (0.8-1.6) |
|  |  | CT | 65 (16.1) | 50 (12.5) | 78 (13.2) | 0.192 | 1.3 (0.9-1.8) | 0.767 | 0.9 (0.6-1.4) |
|  |  | CC | 0 | 2 (0.5) | 6 (1.0) | ------ | ------ | 0.486 | 0.5 (0.1-2.5) |
| GATA3 | rs2280015 | A | 247 (30.6) | 186 (23.3) | 331 (27.8) | 0.171 | 1.1 (0.9-1.4) | 0.022 | 0.8 (0.6-0.9) |
|  |  | GG | 199 (49.4) | 234 (58.5) | 315 (52.9) | 0.269 | 0.9 (0.7-1.1) | 0.084 | 1.3 (1.0-1.6) |
|  |  | AG | 161 (40.0) | 146 (36.5) | 229 (38.5) | 0.642 | 1.1 (0.8-1.4) | 0.526 | 0.9 (0.7-1.2) |
|  |  | AA | 43 (10.7) | 20 (5.0) | 51 (8.6) | 0.265 | 1.3 (0.8-2.0) | 0.032 | 0.6 (0.3-1.0) |
|  | rs3781093 | G | 281 (34.9) | 211 (26.4) | 357 (30.2) | 0.029 | 1.2 (1.0-1.5) | 0.064 | 0.8 (0.7-1.0) |
|  |  | AA | 178 (44.2) | 210 (52.5) | 285 (48.2) | 0.208 | 0.8 (0.7-1.1) | 0.186 | 1.2 (0.9-1.5) |
|  |  | AG | 169 (41.9) | 169 (42.2) | 255 (43.1) | 0.705 | 1.0 (0.7-1.2) | 0.779 | 1.0 (0.7-1.2) |
|  |  | GG | 56 (13.9) | 21 (5.2) | 51 (8.6) | 0.009 | 1.7 (1.1-2.6) | 0.044 | 0.6 (0.3-1.0) |
|  | rs569421 | C | 267 (33.1) | 196 (24.6) | 331 (27.8) | 0.010 | 1.3 (1.1-1.6) | 0.112 | 0.8 (0.7-1.0) |
|  |  | TT | 191 (47.4) | 223 (55.9) | 315 (52.9) | 0.091 | 0.8 (0.6-1.0) | 0.346 | 1.1 (0.9-1.5) |
|  |  | CT | 157 (39.0) | 156 (39.1) | 231 (38.8) | 0.949 | 1.0 (0.8-1.3) | 0.914 | 1.0 (0.8-1.3) |
|  |  | CC | 55 (13.6) | 20 (5.0) | 50 (8.4) | 0.008 | 1.7 (1.2-2.6) | 0.041 | 0.6 (0.3-1.0) |

| **Genes** | **SNPs** | **Allele/Geno** | **BD**  **(frequency)** | **VKH**  **(frequency)** | **Control**  **(frequency)** | **BD**  **P value** | **OR (95%CI)** | **VKH**  **P value** | **OR (95%CI)** |
| --- | --- | --- | --- | --- | --- | --- | --- | --- | --- |
| GATA3 | rs422628 | C | 44 (5.5) | 50 (6.2) | 54 (4.6) | 0.363 | 1.2 (0.8-1.8) | 0.167 | 1.4 (0.9-2.1) |
|  |  | TT | 361 (89.6) | 354 (88.5) | 540 (91.2) | 0.386 | 0.8 (0.5-1.3) | 0.160 | 0.7 (0.5-1.1) |
|  |  | CT | 40 (9.9) | 42 (10.5) | 50 (8.4) | 0.424 | 1.2 (0.8-1.8) | 0.274 | 1.3 (0.8-2.0) |
|  |  | CC | 2 (0.5) | 4 (1.0) | 2 (0.3) | 0.698 | 1.5 (0.2-10.5) | 0.228 | 3.0 (0.5-16.3) |
|  | rs10905284 | C | 374 (46.6) | 333 (41.6) | 527 (44.7) | 0.387 | 1.1 (0.9-1.3) | 0.181 | 0.9 (0.7-1.1) |
|  |  | AA | 122 (30.4) | 129 (32.3) | 187 (31.7) | 0.672 | 0.9 (0.7-1.2) | 0.854 | 1.0 (0.8-1.3) |
|  |  | AC | 184 (45.9) | 209 (52.2) | 279 (47.3) | 0.664 | 0.9 (0.7-1.2) | 0.125 | 1.2 (0.9-1.6) |
|  |  | CC | 95 (23.7) | 62 (15.5) | 124 (21.0) | 0.319 | 1.2 (0.9-1.6) | 0.029 | 0.7 (0.5-1.0) |
| Rorc | rs3790515 | T | 89 (14.8) | 114 (14.3) | 201 (16.9) | 0.252 | 0.9 (0.7-1.1) | 0.112 | 0.8 (0.6-1.0) |
|  |  | CC | 221 (73.7) | 293 (73.4) | 400 (67.5) | 0.057 | 1.4 (1.0-1.8) | 0.044 | 1.3 (1.0-1.8) |
|  |  | CT | 69 (23.0) | 98 (24.6) | 185 (31.2) | 0.010 | 0.7 (0.5-0.9) | 0.023 | 0.7 (0.5-1.0) |
|  |  | TT | 10 (3.3) | 8 (2.0) | 8 (1.3) | 0.046 | 2.5 (1.0-6.5) | 0.421 | 1.5 (0.6-4.0) |
|  | rs1521177 | C | 149 (19.0) | 178 (22.7) | 227 (19.0) | 0.982 | 1.0(0.8-1.3) | 0.048 | 1.2 (1.0-1.6) |
|  |  | AA | 256 (65.3) | 231 (58.9) | 395 (66.3) | 0.753 | 1.0(0.7-1.3) | 0.019 | 0.7 (0.6-1.0) |
|  |  | AC | 123 (31.4) | 144 (36.7) | 175 (29.4) | 0.500 | 1.1 (0.8-1.5) | 0.015 | 1.4 (1.1-1.8) |
|  |  | CC | 13 (3.3) | 17 (4.3) | 26 (4.4) | 0.409 | 0.8 (0.4-1.5) | 0.985 | 1.0 (0.5-1.9) |
|  | rs9017 | A | 233 (39.0) | 290 (36.4) | 439 (37.0) | 0.353 | 1.1 (0.9-1.4) | 0.791 | 1.0 (0.8-1.2) |
|  |  | GG | 112 (37.5) | 172 (43.2) | 233 (39.3) | 0.596 | 0.9 (0.7-1.2) | 0.218 | 1.2 (0.9-1.5) |
|  |  | AG | 141 (47.2) | 162 (40.7) | 281 (47.4) | 0.948 | 1.0 (0.8-1.3) | 0.020 | 1.3 (1.0-1.7) |
|  |  | AA | 46 (15.4) | 64 (16.1) | 79 (13.3) | 0.402 | 1.2 (0.8-1.8) | 0.226 | 1.2 (0.9-1.8) |

**Supplementary Table 3 (continued 2)**

**Supplementary Table 3 (continued 3)**

| **Genes** | **SNPs** | **Allele/Geno** | **BD**  **(frequency)** | **VKH**  **(frequency)** | **Control**  **(frequency)** | **BD**  **P value** | **OR (95%CI)** | **VKH**  **P value** | **OR (95%CI)** |
| --- | --- | --- | --- | --- | --- | --- | --- | --- | --- |
| Rorc | rs939595 | A | 139 (18.1) | 131 (16.7) | 219 (18.2) | 0.932 | 1.0 (0.8-1.3) | 0.365 | 0.9 (0.7-1.1) |
|  |  | AA | 11 (2.9) | 13 (3.3) | 24 (4.0) | 0.348 | 0.7 (0.3-1.5) | 0.573 | 0.8 (0.4-1.6) |
|  |  | AC | 117 (30.5) | 105 (26.7) | 171 (28.5) | 0.109 | 0.8 (0.6-1.1) | 0.540 | 0.9 (0.7-1.2) |
|  |  | CC | 256 (66.7) | 275 (70.0) | 405 (67.5) | 0.786 | 1.0(0.7-1.3) | 0.412 | 1.1 (0.9-1.5) |
|  | rs949969 | T | 240 (40.1) | 321 (40.1) | 481 (40.6) | 0.864 | 1.0 (0.8-1.2) | 0.848 | 1.0 (0.8-1.2) |
|  |  | CC | 104 (34.8) | 142 (35.5) | 224 (37.8) | 0.382 | 0.9 (0.7-1.2) | 0.466 | 0.9 (0.7-1.2) |
|  |  | CT | 150 (50.2) | 195 (48.7) | 257 (43.3) | 0.053 | 1.3 (1.0-1.7) | 0.093 | 1.2 (1.0-1.6) |
|  |  | TT | 45 (15.1) | 63 (15.8) | 112 (18.9) | 0.155 | 0.8 (0.5-1.1) | 0.203 | 0.8 (0.6-1.1) |
|  | rs11204894 | T | 74 (12.3) | 100 (12.5) | 144 (12.1) | 0.897 | 1.0 (0.8-1.4) | 0.785 | 1.0 (0.8-1.4) |
|  |  | GG | 231 (77.0) | 307 (76.9) | 457 (76.9) | 0.983 | 1.0 (0.7-1.4) | 0.998 | 1.0 (0.7-1.4) |
|  |  | GT | 64 (21.3) | 84 (21.1) | 130 (21.9) | 0.850 | 1.0 (0.7-1.4) | 0.754 | 1.0 (0.7-1.3) |
|  |  | TT | 5 (1.7) | 8 (2.0) | 7 (1.2) | 0.549 | 1.4 (0.4-4.5) | 0.295 | 1.7 (0.6-4.8) |
|  | rs11588258 | T | 77 (10.0) | 74 (9.6) | 127 (11.0) | 0.499 | 0.9 (0.7-1.2) | 0.349 | 0.9 (0.6-1.2) |
|  |  | AA | 311 (80.8) | 313 (81.5) | 460 (79.4) | 0.613 | 1.1 (0.8-1.5) | 0.431 | 1.1 (0.8-1.6) |
|  |  | AT | 71 (18.4) | 68 (17.7) | 111 (19.2) | 0.777 | 1.0 (0.7-1.3) | 0.568 | 0.9 (0.7-1.3) |
|  |  | TT | 3 (0.8) | 3 (0.8) | 8 (1.4) | 0.388 | 0.6 (0.1-2.1) | 0.391 | 0.6 (0.1-2.1) |
|  | rs12045886 | C | 358 (44.4) | 361 (45.5) | 539 (45.5) | 0.626 | 1.0 (0.8-1.2) | 0.979 | 1.0 (0.8-1.2) |
|  |  | TT | 125 (31.0) | 110 (27.7) | 189 (31.9) | 0.762 | 1.0 (0.7-1.3) | 0.157 | 0.8 (0.6-1.1) |
|  |  | CT | 198 (49.1) | 213 (53.7) | 267 (45.1) | 0.211 | 1.2 (0.9-1.5) | 0.008 | 1.4 (1.1-1.8) |
|  |  | CC | 80 (19.9) | 74 (18.6) | 136 (23.0) | 0.241 | 0.8 (0.6-1.1) | 0.102 | 0.8 (0.6-1.1) |

**Supplementary Table 3 (continued 4)**

| **Genes** | **SNPs** | **Allele/Geno** | **BD**  **(frequency)** | **VKH**  **(frequency)** | **Control**  **(frequency)** | **BD**  **P value** | **OR (95%CI)** | **VKH**  **P value** | **OR (95%CI)** |
| --- | --- | --- | --- | --- | --- | --- | --- | --- | --- |
| Rorc | rs3828057 | T | 223 (27.7) | 193 (24.1) | 283 (23.8) | 0.050 | 1.2 (1.0-1.5) | 0.860 | 1.0 (0.8-1.3) |
|  |  | CC | 220 (54.6) | 242 (60.5) | 341 (57.3) | 0.395 | 0.9 (0.7-1.2) | 0.317 | 1.1 (0.9-1.5) |
|  |  | CT | 143 (35.5) | 123 (30.7) | 225 (37.8) | 0.454 | 0.9 (0.7-1.2) | 0.022 | 0.7 (0.6-1.0) |
|  |  | TT | 40 (9.9) | 35 (8.7) | 29 (4.9) | 0.002 | 2.2 (1.3-3.5) | 0.015 | 1.9 (1.1-3.1) |
|  | rs7540530 | A | 311 (41.6) | 331 (43.7) | 490 (42.5) | 0.703 | 1.0 (0.8-1.2) | 0.602 | 1.1 (0.9-1.3) |
|  |  | AA | 57 (15.2) | 64 (16.9) | 84 (14.6) | 0.772 | 1.1 (0.7-1.5) | 0.330 | 1.2 (0.8-1.7) |
|  |  | AG | 197 (52.7) | 203 (53.6) | 322 (55.8) | 0.343 | 0.9 (0.7-1.1) | 0.495 | 0.9 (0.7-1.2) |
|  |  | GG | 120 (32.1) | 112 (29.6) | 171 (29.6) | 0.423 | 1.1 (0.8-1.5) | 0.978 | 1.0 (0.8-1.3) |
|  | rs9826 | G | 187 (23.3) | 229 (28.7) | 323 (27.1) | 0.058 | 0.8 (0.7-1.0) | 0.435 | 1.1 (0.9-1.3) |
|  |  | AA | 229 (57.1) | 206 (51.6) | 319 (53.5) | 0.265 | 1.2 (0.9-1.5) | 0.557 | 0.9 (0.7-1.2) |
|  |  | AG | 157 (39.2) | 157 (39.3) | 231 (38.8) | 0.900 | 1.0 (0.8-1.3) | 0.852 | 1.0 (0.8-1.3) |
|  |  | GG | 15 (3.7) | 36 (9.0) | 46 (7.7) | 0.010 | 0.5 (0.3-0.8) | 0.463 | 1.2 (0.8-1.9) |

Bonferroni correction for the number of genotypes tested by the conditional analysis, P value less than 0.05/75=0.0007 was supposed significant.

Bonferroni correction for the number of minor alleles tested by the conditional analysis, P value less than 0.05/28=0.0018 was supposed significant.

**Supplementary Table 4 Summary of the association of Foxp3SNPs with male patients with Behcet’s disease, VKH syndrome** in Chinese Han population

| **Genes** | **SNPs** | **Allele/Geno** | **BD**  **(frequency)** | **VKH**  **(frequency)** | **Control**  **(frequency)** | **BD**  **P value** | **OR (95%CI)** | **VKH**  **P value** | **OR (95%CI)** |
| --- | --- | --- | --- | --- | --- | --- | --- | --- | --- |
| Foxp3 | rs3761549 | C/- | 255 (77.3) | 162 (77.2) | 234 (80.7) | 0.298 | 0.8 (0.6-1.2) | 0.335 | 0.8 (0.5-1.2) |
|  |  | T/- | 75 (22.7) | 48 (22.8) | 56 (19.3) | 0.298 | 1.2 (0.8-1.8) | 0.335 | 1.2 (0.8-1.9) |
|  | rs3761548 | A/- | 74 (22.4) | 48 (22.8) | 60 (20.7) | 0.601 | 1.1 (0.8-1.6) | 0.561 | 1.1 (0.7-1.7) |
|  |  | C/- | 256 (77.6) | 162 (77.2) | 230 (79.3) | 0.601 | 0.9 (0.6-1.3) | 0.561 | 0.9 (0.6-1.4) |
|  | rs2232365 | A/- | 177 (53.6) | 112 (53.3) | 174 (60.6) | 0.080 | 0.8 (0.5-1.0) | 0.104 | 0.7 (0.5-1.1) |
|  |  | G/- | 153 (46.4) | 98 (46.7) | 113 (39.4) | 0.080 | 1.3 (1.0-1.8) | 0.104 | 1.3 (0.9-1.9) |

Bonferroni correction for the number of minor alleles tested by the conditional analysis, P value less than 0.05/28=0.0018 was supposed significant.

**Supplementary Table 5 Summary of the association of FOXP3SNPs with in female patients with Behcet’s disease, VKH syndrome** in Chinese Han population

| **Genes** | **SNPs** | **Allele/Geno** | **BD**  **(frequency)** | **VKH**  **(frequency)** | **Control**  **(frequency)** | **BD**  **P value** | **OR (95%CI)** | **VKH**  **P value** | **OR (95%CI)** |
| --- | --- | --- | --- | --- | --- | --- | --- | --- | --- |
| Foxp3 | rs3761549 | T | 33 (22.6) | 77 (20.4) | 119 (19.5) | 0.402 | 1.2 (0.8-1.9) | 0.741 | 1.1 (0.8-1.5) |
|  |  | CC | 46 (63.0) | 121 (64.0) | 202 (66.2) | 0.603 | 0.9 (0.5-1.5) | 0.616 | 0.9 (0.6-1.3) |
|  |  | CT | 21 (28.8) | 59 (31.2) | 87 (28.5) | 0.967 | 1.0 (0.6-1.8) | 0.524 | 1.1 (0.8-1.7) |
|  |  | TT | 6 (8.2) | 9 (4.8) | 16 (5.3) | 0.330 | 1.6 (0.6-4.3) | 0.811 | 0.9 (0.4-2.1) |
|  | rs3761548 | A | 37 (25.3) | 78 (20.6) | 123 (20.1) | 0.162 | 1.3 (0.9-2.1) | 0.838 | 1.0 (0.8-1.4) |
|  |  | CC | 40 (54.8) | 123 (65.1) | 193 (63.1) | 0.192 | 0.7 (0.4-1.2) | 0.652 | 1.1 (0.7-1.6) |
|  |  | AC | 29 (39.7) | 54 (28.6) | 103 (33.7) | 0.328 | 1.3 (0.8-2.2) | 0.237 | 0.8 (0.5-1.2) |
|  |  | AA | 4 (5.5) | 12 (6.3) | 10 (3.3) | 0.368 | 1.7 (0.5-5.6) | 0.106 | 2.0 (0.9-4.7) |
|  | rs2232365 | G | 67 (45.9) | 165 (43.7) | 250 (41.1) | 0.294 | 1.2 (0.8-1.7) | 0.433 | 1.1 (0.9-1.4) |
|  |  | AA | 26 (35.6) | 57 (30.2) | 104 (34.2) | 0.820 | 1.1 (0.6-1.8) | 0.351 | 0.8 (0.6-1.2) |
|  |  | AG | 27 (37.0) | 99 (52.4) | 150 (49.3) | 0.057 | 0.6 (0.4-1.0) | 0.512 | 1.1 (0.8-1.6) |
|  |  | GG | 20 (27.4) | 33 (17.5) | 50 (16.4) | 0.031 | 1.9 (1.1-3.5) | 0.770 | 1.1 (0.7-1.7) |

Bonferroni correction for the number of genotypes tested by the conditional analysis, P value less than 0.05/75=0.0007 was supposed significant.

Bonferroni correction for the number of minor alleles tested by the conditional analysis, P value less than 0.05/28=0.0018 was supposed significant.

**Supplementary Table 6 Variation of allele frequencies between Han Chinese population and Caucasian population**

| **Genes** | **SNPs** | **Allele** | **Frequencies of Chinese** | **Known Frequencies of Caucasion** | **Chi^2^** | **P value** |
| --- | --- | --- | --- | --- | --- | --- |
|
| TBX21 | rs7502875 | A/C | 0.943/0.057 | 0.783/0.217 | 49.99 | 1.54×10^-12^ |
|  | rs10514934 | C/T | 0.131/0.869 | 0.133/0.867 | 0.003 | 0.995 |
|  | rs16947058 | A/G | 0.194/0.806 | 0.438/0.562 | 63.05 | 2.01×10^-15^ |
|  | rs2074190 | C/T | 0.099/0.901 | 0.239/0.761 | 35.1 | 3.13×10^-9^ |
|  | rs4794067 | C/T | 0.121/0.879 | 0.239/0.761 | 55.64 | 8.70×10^-14^ |
|  | rs17250932 | C/T | 0.076/0.924 | -/- | - | - |
| GATA3 | rs2280015 | A/G | 0.278/0.722 | 0.159/0.841 | 13.98 | 1.85×10^-4^ |
|  | rs3781093 | A/G | 0.698/0.302 | 0.878/0.122 | 30.61 | 3.15×10^-8^ |
|  | rs569421 | C/T | 0.278/0.722 | 0.204/0.796 | 5.35 | 0.021 |
|  | rs422628 | C/T | 0.046/0.954 | 0.248/0.752 | 107.85 | 2.90×10^-25^ |
|  | rs10905284 | A/C | 0.553/0.447 | 0.584/0.416 | 0.724 | 0.395 |
| Rorc | rs3790515 | C/T | 0.831/0.169 | 0.956/0.044 | 23.42 | 1.30×10^-6^ |
|  | rs1521177 | A/C | 0.810/0.190 | 0.482/0.518 | 110.74 | 6.76×10^-26^ |
|  | rs9017 | A/G | 0.370/0.630 | 0.597/0.403 | 40.61 | 1.86×10^-10^ |
|  | rs939595 | A/C | 0.182/0.818 | 0.407/0.593 | 56.25 | 6.39×10^-14^ |
|  | rs949969 | C/T | 0.594/0.406 | 0.739/0.261 | 16.78 | 4.19×10^-5^ |
|  | rs11204894 | G/T | 0.879/0.121 | 0.841/0.159 | 2.48 | 0.115 |
|  | rs11588258 | A/T | 0.890/0.11 | 0.920/0.080 | 1.82 | 0.178 |
|  | rs12045886 | C/T | 0.455/0.545 | 0.332/0.668 | 11.75 | 0.001 |
|  | rs3828057 | C/T | 0.762/0.238 | 0.580/0.420 | 32.34 | 1.29×10^-8^ |
|  | rs7540530 | A/G | 0.425/0.575 | 0.535/0.465 | 9.40 | 0.002 |
|  | rs9826 | A/G | 0.729/0.271 | 0.608/0.392 | 13.29 | 2.67×10^-4^ |
| Foxp3 | rs3761549 | C/T | 0.806/0.194 | 0.845/0.155 | 1.87 | 0.172 |
|  | rs3761548 | A/C | 0.203/0.797 | 0.412/0.588 | 42.56 | 6.86×10^-11^ |
|  | rs2232365 | A/G | 0.594/0.406 | 0.434/0.566 | 18.44 | 1.76×10^-5^ |

Bonferroni correction for the number of allele frequencies tested by the conditional analysis, P value less than 0.05/24=0.002 was supposed significant.

**Supplementary Table 7 Primers used in MassARRAY® platform (Sequenom)**

| **SNP** | **Forward Primer** | **Reverse Primer** | **Primer for Single Base Extension** |
| --- | --- | --- | --- |
| rs7502875 | ACGTTGGATGTCTGATCCCCACTGTGTTTG | ACGTTGGATGTGTTATAACCATCAGCCCGC | GGGTCAGGGAAAGGACTC |
| rs16947058 | ACGTTGGATGGATGGGATGTGAACTGTGAC | ACGTTGGATGTTGGGTAGTTCTCCCAGTTC | CCCCTTTCTCCCAGTTCTGTTTAA |
| rs2074190 | ACGTTGGATGGTGAGGACTACGCGCTACC | ACGTTGGATGGCTGATTAAACTTGGACCAC | TTCCCCGACACCTCCAG |
| rs4794067 | ACGTTGGATGAGGCAGAAACTTCCCTGTTC | ACGTTGGATGGACAGTACCAGAAACACAGG | TGGCCTCCTCCCCAACACCTTACCC |
| rs17250932 | ACGTTGGATGATAAAGCAGCATGTGTAGTG | ACGTTGGATGCTCTGAGACCTCACTCCTTA | GTCTCTGTCTTTTGCACAC |
| rs2280015 | ACGTTGGATGAGCCATCAGGATGCATTCAC | ACGTTGGATGATCCAGAGTGTTTTTGGTGC | TTTTGGTGCTTGGCTTTGC |
| rs3781093 | ACGTTGGATGTCCGCTGTCTCTCTACAGAT | ACGTTGGATGTCAAGGCTGCTCATCTCATC | GGGTTTTCACGATTGCTTTCT |
| rs569421 | ACGTTGGATGTATGAGTCTTCCCTTCGGTC | ACGTTGGATGAGCCCATATATAGCTAGCTC | TAGCTAGCTCTTCTGTGAGATG |
| rs422628 | ACGTTGGATGTGCTCTCCTGGCTGCAGAC | ACGTTGGATGAGCCTGTCTTCATAGTGATG | CACATTTAACATTTGTTTTGATTT |
| rs10905284 | ACGTTGGATGCCTGGGGAGTACTTTTCAAG | ACGTTGGATGGTGAAGAATCTTCAGGAGGG | AGGGAAATGCCATTTTG |
| rs3790515 | ACGTTGGATGATACAGCTTTGCCCTCCAAC | ACGTTGGATGTTGGGCATAACCTTTGTGGG | TTATGTCCTGTTCACCTCAAC |
| rs9017 | ACGTTGGATGTGTGATCTTGCCCAGAACCT | ACGTTGGATGTCCACGACTGCCCATCATTG | CCATCATTGCTGTTAATCC |
| rs949969 | ACGTTGGATGACCTCTGATGATAACTCACC | ACGTTGGATGTTCTCCCCAATCTGAGTTCC | GCCTGCTGCTTCAGGGACGTGTCTCC |
| rs11204894 | ACGTTGGATGAGATGCAGAAAGCCATAGTC | ACGTTGGATGAAAAGGAGAGACAAATGCCC | AAATGCCCAAATGCACCCGCCA |
| rs12045886 | ACGTTGGATGATCCGGTTGGATCTGCTTTC | ACGTTGGATGGAAAGCTGAGAGCTGGTAGG | CGGGCCAGGGTGGTGGTAATAG |
| rs3828057 | ACGTTGGATGAGGGAGGGTCAATACTTCAG | ACGTTGGATGCAAGGAGGGTTTGTCCTAGC | ACCTAGCCCAGGAAGAAT |
| rs9826 | ACGTTGGATGAGAGAAGCAGAAGTCGCTCG | ACGTTGGATGTTCTCCACAACAAGAGCAGG | CGTCCTCCAGGATCTGATCTTGCC |
| rs3761548 | ACGTTGGATGTGGGTGCTGAGGGGTAAACT | ACGTTGGATGAAGCCTAGATCTCAGGACTC | GGGCTCTCTCCCCAACTG |
| rs2232365 | ACGTTGGATGAGGAGTGTGATCATGCACGG | ACGTTGGATGGTAGAGAAGCTTCTACAGGC | CTACAGGCCCCAGCTCAAGAGACCCC |

**Supplementary Table 8 Primers and restriction enzymes used in PCR-RFLP analysis**

| **SNP** | **Primers** | **Tm for PCR** | **Restriction** | **Resriction** |
| --- | --- | --- | --- | --- |
|  |  | **(℃)** | **Enzymes** | **Sites** |
| rs939595 | F:5'CTGAATTGTTCCCTGGAGGT3' | 56 | HinfI | G^ANTC |
|  | R:5'AGGAGGGAGTGCGGGAGTACGA3' |  |  |  |
| rs1521177 | F:5'ATGGATTTGAAGGAGGAGAGA3' | 58 | BseGI (FokI) | GGATGN_9_^ |
|  | R:5'GCCTCTCTTAAAGTCCAAGTGAA3' |  |  |  |
| rs3761549 | F:5'CCGGCTTCCTGCACTGTCTGT 3' | 60 | AluI | AG^CT |
|  | R:5'GGGCCAGGTCTTCAGAGCTAG 3' |  |  |  |
| rs7540530 | F:5'GCGGTTGGGCCAGGGTCATA 3' | 60 | Eco88I | C^YCGRG |
|  | R:5'GGCCTCCTGTGGTCCCATGAAG 3' |  |  |  |
| rs10514934 | F:5'CGGGGGGAGATTGGGATTTG 3' | 60 | Taq I | T^CGA |
|  | R:5'GCCCCAAACTCTCCCCATCAC 3' |  |  |  |
| rs11588258 | F:5'TGCCTTCCCACCCTAGATTTC 3' | 60 | MboI | ^GATC |
|  | R:5'AGGCAATGGCAGGCAAGAGAC 3' |  |  |  |
